# Health workers’ perceptions and challenges in implementing meningococcal serogroup a conjugate vaccine in the routine childhood immunization schedule in Burkina Faso

**DOI:** 10.1186/s12889-020-8347-z

**Published:** 2020-02-19

**Authors:** Sylvain F. Nkwenkeu, Mohamed F. Jalloh, Jenny A. Walldorf, Robert L. Zoma, Felix Tarbangdo, Soukeynatou Fall, Sansan Hien, Roland Combassere, Cesaire Ky, Ludovic Kambou, Alpha Oumar Diallo, Akshaya Krishnaswamy, Flavien H. Aké, Cynthia Hatcher, Jaymin C. Patel, Isaïe Medah, Ryan T. Novak, Terri B. Hyde, Heidi M. Soeters, Imran Mirza

**Affiliations:** 1 UNICEF Ouagadougou, 01 PO Box 3420, Ouagadougou 01, Burkina Faso; 2 U.S. Centers for Disease Control and Prevention, Atlanta, GA 30329 USA; 3 Institut National de Statistique et Démographie, Ouagadougou, Burkina Faso; 4 Davycas International, Ouagadougou, Burkina Faso; 5 Ministère de la Santé, Ouagadougou, Burkina Faso; 6 UNICEF, New York, NY 10017 USA

**Keywords:** Meningococcal vaccine, Measles vaccine, Vaccine introduction, Health workers, Burkina Faso, Vaccination barriers, Perceptions, Challenges, Vaccination demand, Effective vaccine administration, Vaccination coverage improvement, Second year of life, Co-administration

## Abstract

**Background:**

Meningococcal serogroup A conjugate vaccine (MACV) was introduced in 2017 into the routine childhood immunization schedule (at 15–18 months of age) in Burkina Faso to help reduce meningococcal meningitis burden. MACV was scheduled to be co-administered with the second dose of measles-containing vaccine (MCV2), a vaccine already in the national schedule. One year following the introduction of MACV, an assessment was conducted to qualitatively examine health workers’ perceptions of MACV introduction, identify barriers to uptake, and explore opportunities to improve coverage.

**Methods:**

Twelve in-depth interviews were conducted with different cadres of health workers in four purposively selected districts in Burkina Faso. Districts were selected to include urban and rural areas as well as high and low MCV2 coverage areas. Respondents included health workers at the following levels: regional health managers (*n* = 4), district health managers (*n* = 4), and frontline healthcare providers (*n* = 4). All interviews were recorded, transcribed, and thematically analyzed using qualitative content analysis.

**Results:**

Four themes emerged around supply and health systems barriers, demand-related barriers, specific challenges related to MACV and MCV2 co-administration, and motivations and efforts to improve vaccination coverage. Supply and health systems barriers included aging cold chain equipment, staff shortages, overworked and poorly trained staff, insufficient supplies and financial resources, and challenges with implementing community outreach activities. Health workers largely viewed MACV introduction as a source of motivation for caregivers to bring their children for the 15- to 18-month visit. However, they also pointed to demand barriers, including cultural practices that sometimes discourage vaccination, misconceptions about vaccines, and religious beliefs. Challenges in co-administering MACV and MCV2 were mainly related to reluctance among health workers to open multi-dose vials unless enough children were present to avoid wastage.

**Conclusions:**

To improve effective administration of vaccines in the second-year of life, adequate operational and programmatic planning, training, communication, and monitoring are necessary. Moreover, clear policy communication is needed to help ensure that health workers do not refrain from opening multi-dose vials for small numbers of children.

## Background

*Neisseria meningitidis* (Nm) is one of the major global etiologies of meningitis and septicemia [1]. Nm has 12 serogroups, based on the capsular polysaccharide, of which the serogroups A, B, C, W, Y, and X cause life-threatening invasive disease and are frequently implicated in epidemics or outbreaks of meningococcal meningitis [2]. Serogroup A has historically been the cause of epidemic meningitis in sub-Saharan Africa and predominantly affects people < 30 years of age [3]. Infected persons usually experience sudden onset of high fever, neck stiffness, confusion, nausea, and vomiting; about half will die without prompt medical treatment [4]. To help reduce the meningococcal meningitis burden in endemic African countries, meningococcal serogroup A conjugate vaccine (MACV, MenAfriVac™) was introduced beginning in 2010 through mass vaccination campaigns for people from age 1 to 29 years [5]. The incidence of serogroup A meningitis declined sharply following MACV mass vaccination campaigns in these countries, including Burkina Faso [6]. High community acceptance of MACV was reported during the campaigns [7] and resulted in nearly 100% coverage of MACV, based on administrative data from the campaigns.

The World Health Organization (WHO) recommends that countries completing mass vaccination campaigns introduce MACV into the routine childhood immunization schedule within 1–5 years following the campaign, along with a one-time catch-up campaign among children 1 to 6 years of age who were born after the initial campaigns [8]. In 2016, a catch-up campaign was conducted in Burkina Faso and in 2017 MACV was introduce into the routine childhood immunization schedule (which is given both at local health facilities and during outreach activities) at 15 to 18 months of age, the same age at which the second dose of measles-containing vaccine (MCV2) is given [4].

Although MCV2 was introduced into the routine childhood immunization schedule in 2013, national MCV2 coverage in Burkina Faso was estimated to be only 50% in 2017 [9]. MCV2 coverage in Burkina Faso is lower than coverage for the first dose of MCV (MCV1) and other vaccines given during the first year of life because of high dropout rates (i.e., the proportion of children who start but do not complete the vaccination series) [10]. The high community demand observed for MACV during mass vaccination campaigns was seen as a way to improve MCV2 uptake because both vaccines are co-administered at the same visit.

Multiple supply- and demand-side factors impact access to and acceptance of vaccines [11]. Known obstacles to achieving optimal childhood vaccination coverage include operational and logistical factors in effectively transporting, managing, and storing vaccines, vaccine supply shortages, inadequate number of health workers, sociocultural influences such as religious beliefs, and parental knowledge and normative attitudes [12]. A limited number of studies have examined barriers to vaccination uptake in Burkina Faso, especially during the second year of life (12 to 23 months of age) [13–17].

One-year following the introduction of MACV into the routine childhood immunization schedule, we aimed to qualitatively examine health workers’ perceptions of MACV introduction, identify barriers to uptake, and explore opportunities to improve coverage. The qualitative assessment was conducted concurrently with a quantitative nationwide MACV/MCV vaccination coverage survey, which is reported elsewhere [18]. This paper focuses on the qualitative assessment conducted with health workers, from frontline healthcare providers to Expanded Program on Immunization (EPI) managers at the district and regional levels. It brings novel and important information on barriers to vaccination uptake in Burkina Faso, especially during the second year of life, from the health workers’ perspectives. The paper provides recommendations to improve effective administration of vaccines in the second-year of life, based on system and demand-related barriers and challenges related to co-administration of MACV and MCV2 in Burkina Faso.

## Methods

### Study design

Based on principles of stakeholder analysis approach [19–21], twelve in-depth interviews (IDIs) were conducted across four geographic regions in Burkina Faso, comprising both rural and urban areas in four districts purposefully selected based on 2016 administrative MCV2 coverage (Table 1). Two districts with low coverage (Koupela and Baskuy, < 50% MCV2 coverage) and two districts with high coverage (Mangodara and Ouahigouya, > 90% MCV2 coverage) were purposively selected based on administrative data collected by the Burkina Faso Ministry of Health.

**Table 1:** Distribution of in-depth interviews by district, MCV2 coverage, and geographic setting, qualitative assessment survey of healthcare workers, Burkina Faso, 2018

District	MCV2^a^ coverage	Geographic Setting	Interviews conducted
Mangodara	High	Rural	3
Ouahigouya		Urban	3
Koupela	Low	Rural	3
Baskuy		Urban	3
Total			12

^a^*MCV2* second dose of measles-containing vaccine

### Sampling procedures and data collection

For each of the four selected districts, interviews were conducted with three cadres of EPI workers: regional EPI managers (representing the district), district EPI managers, and frontline healthcare providers. Regional and district EPI managers were identified based on their designated titles, given that the role is fulfilled by only one staff member at the respective levels across regions and districts. At the health facility level, healthcare providers who were directly responsible for providing childhood vaccination services were purposively selected.

Technical experts from the Burkina Faso Ministry of Health (MoH), Davycas International (local non-governmental organization), the United Nations Children’s Fund (UNICEF), and the US Centers for Disease Control and Prevention (CDC) developed an interview guide (Supplemental Material 1). The interview guide covered six areas: perceptions of meningitis and measles, information sources about childhood vaccination, challenges and motivations for caregivers to bring their children to the 15-month visit, barriers to vaccination in general and specifically regarding co-administration of MACV and MCV2, and recommendations to improve uptake of vaccines. Each domain included several open-ended questions along with suggested probes and follow-up questions. The interview guides were pilot tested following the training of data collectors, and modifications were made to improve understanding of questions and clarity of probes.

Overseen by qualitative evaluation experts from UNICEF and CDC, four male data collectors from Davycas International and two supervisors from the MoH were trained to conduct all aspects of the qualitative assessment. Data collectors were all nationals of Burkina Faso possessing at least bachelor’s level degrees while supervisors possessed master’s level degrees in social sciences or public health. A 1-week qualitative methods training covered selection of respondents, informed consent, qualitative interviewing techniques, proper administration of the interview guide, data management, transcription, and quality control. The training was followed by field testing of the interview guide, which then informed revisions to the guide to improve understanding for local communities, relevance, and order of questions and probes.

Data collection took place during February and March 2018 and was conducted by teams comprised of an interviewer and a note-taker, all of whom were from Burkina Faso and had prior experience in qualitative methodologies. Three regional supervisors involved in the quantitative vaccination coverage survey performed quality control checks with the data collection teams [18]. Each team interviewed two respondents each day in their local language (or French in some instances) at their designated health facilities or office locations; on average, each interview lasted approximately between 45 and up to 90 min. All 12 respondents provided written informed consent, and the interviews were audio-recorded with their permission. None refused to consent. No one else was present during the interview besides the interviewer and note-taker. At the end of each interview, the data collection team used a structured template to debrief on their observations, interview dynamics, and contextual issues (Supplemental Material 2). Repeat interviews were not conducted. Data saturation were continuously assessed by reviewing the daily debriefing notes. We determined that saturation was achieved with the 12 interviews, which was further confirmed during the content analysis of transcripts.

### Data processing and analysis

Audio recordings of the interviews were transcribed by the teams that conducted the interviews. In total, the audio recordings resulted in more than 200 pages of transcribed text in Microsoft Word (version 2016). To ensure consistency and accuracy of transcriptions, the three regional supervisors reviewed 50% of all audio recordings against the final transcripts. Discrepancies were subsequently discussed and resolved in consultations between data collection teams and supervisors.

Qualitative content analysis was used to analyze the data via an iterative process that began with a full reading of all notes and transcripts by three team members with data advanced qualitative analysis skills; all had extensive experience with the local sociocultural environment in Burkina Faso. A careful review of the transcripts, interview notes, and the interview guide informed the development of a thematic codebook (Supplemental Material 3, in French) used to organize the data. All transcripts were uploaded into Dedoose, a web-based qualitative analysis software [22], where excerpts from the transcripts were first coded based on the established codebook. Any new inductive codes that emerged from the transcripts were added to the codebook. At the end of the coding process, the analysts reviewed each other’s application of codes, and the codes were then categorized. Attribution of meaning and interpretations when applying codes and categorizing themes were reviewed and harmonized among the analysts. The emergent categories of codes were subsequently organized into themes.

### Ethical considerations

The assessment protocol was approved by the Ethics Committee for Health Research in Burkina Faso. This project was reviewed in accordance with CDC human research protection procedures and was determined to be non-research, routine public health activity not requiring CDC Institutional Review Board review.

## Results

Based on the structured IDIs, the four major themes that emerged were related to supply and health systems barriers, demand-related barriers, specific challenges related to MACV and MCV2 co-administration, and motivations and efforts to improve vaccination coverage. These themes were crosscutting, and no meaningful differences were observed between high and low MCV2 coverage areas or between rural and urban areas.

### Supply and health systems barriers

Aging cold chain equipment was frequently raised as a logistical barrier to maintaining an adequate supply of vaccines at the local health facility level. In addition, interviewees mentioned the need to provide periodic training to health facility staff on cold chain management. Respondents stated that they often resort to storing vaccines centrally at the district level, where the refrigerators are reliably functional. In addition, they shared their opinion that staff shortages lead to overwork among health workers who are responsible for wide-ranging tasks, including nursing care, registering children, filing records, managing and administering vaccines, and health education.

*“You will agree with me that I am alone in this health facility and there is a lot to do. If there are several activities underway, I cannot leave for [vaccination] outreach activities. Today I had to consult patients and do the prenatal consultation alone, while there are several documents to fill. It's quite cumbersome to manage.”* – Frontline healthcare provider

Having insufficient supplies and financial resources to conduct vaccination outreach activities was another logistical barrier reported. In some cases, respondents shared that they have used their personal finances to conduct community awareness and outreach. Respondents also reported the inaccessibility of some vaccination sites, especially during the rainy season.

One respondent reported that the introduction of a second MCV dose at the 15- to 18-month visit was not preceded by proper training and supportive supervision. Others mistakenly considered MCV2 as a different antigen rather than a second dose of the same vaccine (MCV) administered at 9 months of age.

*“You see, for example, when you say MCV2, even the health workers at first did not understand. We went once on supervision where a vaccinator said he did not administer MCV2 because the vaccine wasn’t there. This is because he did not know the difference between MCV1 and MCV2. It is all about the contact, MCV1 being the first contact and MCV2 the second. He believed that the introduction of MCV2 was another vaccine.” –* EPI Manager at district level

### Perceptions of sociocultural barriers

Both healthcare providers and EPI managers believed their environment is influenced by an array of sociocultural factors that might drive or impede vaccination uptake. They referenced high illiteracy among caregivers, which they believed can lead to poor management of the child’s vaccination card. Some respondents highlighted concerns about cultural practices that they perceived as discouraging vaccination uptake at the community level, including misperceptions of meningitis prevention and misconceptions about the meningitis vaccines. In some cases, respondents believed that caregivers with children close in age would become discouraged to attend vaccination sessions because of the social stigma associated with having closely-spaced children.

“*It often happens that a woman, before completing the vaccination of her child, becomes pregnant. She does not want to come to the gathering place because of other people's eyes—you have not even finished vaccinating your child and then you're pregnant.*” – EPI Manager at district level

Respondents perceived that members in many communities have experienced the benefits of vaccination in preventing devastating childhood diseases, which in turn have influenced their confidence in vaccines in a positive way. In other communities, however, members believed that doubts about vaccines can lead to mistrust. For example, the influence of traditional healers, who are not always supportive of modern medicine, was raised as a source of vaccine hesitancy in some communities:

*“I saw a child who was not up to date with his vaccines. I asked the mom and she told me that the child's dad was a traditional practitioner and does not want his child to be vaccinated.” –* Frontline healthcare provider

*“In order to get their product used, some traditional practitioners tell you, for example, that their product is conflicting with modern medicines. So do not associate traditional products with that of the modern medicine.” –* EPI Manager at district level

Some EPI managers said that pockets of people in communities resist vaccination based on their religious beliefs, especially in isolated areas.

*“Those who refuse is because of their personal belief, not because they mistrust the vaccinator. This is because they are convinced that the vaccine they are receiving is foreign to their body and not recommended by God.” –* EPI Manager at district level

*“For this sect, it is God who gives life, it is God who gives the disease, and it is God who also takes life. So, someone must not do anything to prevent God's will.” –* EPI Manager at district level

Through not widespread, interviewees reported that misconceptions about vaccine safety and effectiveness have been gaining traction in some rural communities where vaccination outreach is often difficult because of long travel distances. Respondents highlighted the need to generally address community-level misconceptions about disease risk and prevention, including for vaccine-preventable diseases.

*“The strategies that can be used are mobilization, sensitization of the mothers through the existing channels and platforms—women’s associations, community-based associations, etc., and awareness-raising and advocacy towards religious leaders. It is necessary to use the public criers as relays in mobilizing community.” –* EPI Manager at district level

Finally, competing priorities among caregivers emerged as another perceived barrier in our sample of health workers at all three levels. Respondents believed that activities related to the livelihood of community members (e.g., petty trading, raising livestock, subsistence farming) prevent some caregivers from returning with their children for scheduled vaccinations.

### Challenges related to MACV and MCV2 co-administration

Packaging of vaccines in 10-dose vials (both for MACV and MCV) that must be discarded 6–8 h after opening was a commonly expressed barrier to co-administering MACV and MCV2. Respondents cited their reluctance to open a 10-dose vaccine vial for fewer children to avoid vaccine wastage or shortages, and they acknowledged that this practice created missed vaccination opportunities and delays for many children:

*“If it's a child or two, you cannot open the 10-dose vial. You have to gather at least 5 children to open it. However, if during a vaccination session we have only 2 or 3 children, we ask the mother to come back the following week, which discourages them.”* – Frontline healthcare provider

*“It is not efficient to open a bottle for any single child. We need a group of children in order to open the bottle. On the other hand, if the children are grouped at the beginning, we know that* a priori *we will not have difficulties to open a bottle since we are about to empty its content.” –* EPI manager at the regional level

Even though there is a policy requiring the opening of a multi-dose vial even for one child, respondents appeared to be worried about vaccine stocks and the close monitoring of vaccine stocks by EPI managers at the regional level, which are part of vaccinators’ performance appraisals. This was mentioned by EPI managers at both district and regional levels because vaccines are supplied based on target population estimates projected from the 2006 census. The same vaccine is required for the first and second dose of measles vaccine as compared to single dose of MACV; therefore, more children may be eligible to receive a dose of MCV than a dose of MACV.

*“Imagine that I have 3 children for MCV1 and 2 for MCV2, which totals 5, the minimum of children required to open my MCV 10-dose vial. On the other hand, this is not enough to open the* MACV *vial as I have only 2 eligible children. Unpackaging it and administering 2 doses entails a loss of 8 doses. In such a situation, what should be done to minimize wastage?” –* EPI manager, district level

According to healthcare providers interviewed, children sometimes experience fever and fatigue following co-administration of MCV and MACV vaccines. They reported that vaccine side effects such as these might sometimes discourage mothers from returning with their children for other scheduled vaccination visits. To minimize side effects, they sometimes opt to administer an analgesic (anti-pain) and antipyretic (anti-fever) to children.

Several respondents noted that some caregivers are concerned about their children receiving multiple injectable vaccines in a single visit.

To address all of these challenges and concerns, health workers pointed to the need to raise caregiver awareness and knowledge regarding the timeliness and importance of the 15- to 18-month visit for MACV and MCV2. Respondents shared their view that improving demand for MACV and MCV2 vaccination will require strengthening caregiver engagement, especially in rural communities where the level of caregiver education remains low.

### Motivations and efforts to improve vaccination coverage

According to respondents, caregivers’ motivations for vaccination revolved around the desire to keep their child healthy. They feared seeing children suffer from meningitis or measles, which mothers recognized as having been the cause of deadly epidemics in the past. Health workers therefore largely viewed caregivers as being inclined to participate in efforts to have their children vaccinated against these diseases.

*“As they are aware of the gravity of meningitis and measles, mothers do not want their children to be affected by these diseases, so they must automatically come to get the vaccination.”–* Frontline healthcare provider

Respondents reported that the introduction of MACV at the 15- to 18-month visit not only has facilitated catch-up of missed children for other vaccines, but also has contributed to improving uptake of MCV2.

*“The introduction of* MACV *into the immunization calendar has brought a plus. When I take our coverage this year for MCV2, for example, we gained almost 10 points, from 56 points last year to 66 points this year.* MACV *… has generated enthusiasm for the population.” –* EPI Manager at district level

Respondents reported that caregivers appreciated the implementation of community outreach activities because it prevents them from travelling long distances to vaccinate their children. For that, healthcare providers said that they relied heavily on support from community leaders and community health workers (CHWs).

Respondents said that providing non-cash incentives to mothers of fully immunized children (such as long-lasting insecticide-treated bed nets) leads mothers to comply with the routine immunization schedule for their children. This practice of incentivizing mothers to fully vaccinate their children was reportedly underway in several health facilities in the districts visited.

*“There are also others who take advantage of mosquito nets that are given to health facilities for children under 5 years old and pregnant women, to motivate mothers during immunization sessions.” –* EPI Manager at district level

## Discussion

These findings reveal various factors that contribute to vaccine uptake after the introduction of MACV in the routine childhood immunization program in Burkina Faso, as well as challenges faced by health workers conducting vaccination programs for children during their second year of life in general. Based on health workers’ perspectives, some factors acted as facilitators of and barriers to service delivery and demand for MACV and MCV2. Respondents frequently cited caregivers’ motivation to bring their children for the 15- to 18-month visit because of the demand for MACV. Recurring service delivery barriers reported by respondents included aging cold chain equipment, resource constraints to conducting community outreach, and reluctance among health workers to open multi-dose vials unless sufficient numbers of children were present in order to avoid wastage. Demand-related barriers included sociocultural beliefs and practices that sometimes discourage vaccination. Taken together, these results point to the need for adequate operational and programmatic planning, health worker training, and clear policy communication regarding the opening of multi-dose vials.

Proper functioning of the cold chain, access equity to ensure reaching hard-to-reach areas, and high-quality service delivery were highlighted by respondents as important supply-side priorities to improve implementation of childhood immunization services at the district level. Other studies in Burkina Faso have documented inadequate vaccination coverage caused by myriad logistical constraints, frequent stock-outs of vaccines, and workforce constraints including staff shortages at sub-district levels [23, 24]. Poor working conditions such as lack of resources to conduct regular outreach services, especially in the more remote areas, coupled with the lack of periodic training on cold chain management, appeared to undermine the motivation of health workers in this sample. In addition, respondents emphasized their inability to engage in health education with caregivers during immunization sessions because they are overloaded with many competing responsibilities, such as registering children, filing records, and managing and administering vaccines. The findings of an overstretched workforce and competing priorities among health workers are consistent with prior studies [25].

Despite not experiencing stock-outs, health workers were reluctant to open the 10-dose vials for fewer children for fear of vaccine wastage or shortages. It is possible that health workers were worried about accountability for their allotted vaccines based on population estimates for children in their catchment geographic areas. The practice of not opening vials unless a certain minimum number of children were present has been observed in other resource-limited settings [26–29]. In an analysis of 46 countries, including Burkina Faso, one study revealed that missed opportunities to vaccinate children with MCV were more frequent than for diphtheria-pertussis-tetanus (DPT) vaccine or oral polio vaccine; this may in part be due to the fact that MCV is a lyophilized vaccine and must be discarded within a 6- to 8-h window once reconstituted or at the end of the immunization session, whichever comes first, whereas DPT and polio vaccines can be used as long as the Vaccine Vial Monitor is valid or the vaccine is not expired [30]. These findings point to the need to emphasize the WHO recommendation [31] to open a multi-dose vial for even one eligible child in order to decrease missed opportunities for vaccination [31]; health worker training should be done for new hires along with in-service training of existing staff to reinforce appropriate practices [32].

A previous study in Burkina Faso found that parental knowledge of the preventive benefit of immunization was associated with complete immunization status in rural areas [33]. Another study demonstrated that sociodemographic factors, such as mothers’ education and area of residence, were associated with lower vaccination coverage [34]. In Burkina Faso and elsewhere, various efforts continue to address parental/caregiver concerns regarding vaccines, including fear of adverse events following immunization. In addition to demand-related barriers, inequities to access remain significant, large pockets of low vaccination coverage persist, and coverage varies considerably across regions, districts, and health facilities’ catchment areas [35].

In a nationwide coverage survey one year after the introduction of MACV in the routine childhood immunization schedule, results suggest a small increase (almost 5%) in MCV2 coverage compared with the period before MACV introduction [18]. However, given the methodology of the survey, such an increase in coverage cannot be statistically attributed to MACV introduction. In the same survey, the most frequently cited reasons by caregivers for not vaccinating their children were lack of awareness of the 15- to 18-month EPI visit and vaccine unavailability.

Interactions between caregivers and health workers during immunization sessions were considered to be very important factors for complete vaccination, according to health workers in our assessment. This sample of health workers also believed that caregivers’ low education levels and illiteracy were having a negative effect on timely adherence to completing the vaccination schedule, confirming findings from previous studies [36, 37]. Even though respondents acknowledged that most caregivers know the disease prevention benefits of immunization, uneducated caregivers must be reminded about their appointments because of their inability to read their child health record cards.

According to respondents in this study, only few and isolated cases of active vaccination refusal, which were largely attributed to religious beliefs, have occurred. These results also indicate that passive refusal may stem from other underlying mistrust of authorities, coupled with personal beliefs and the influence of local traditional healers. Taken together, these factors may lead to vaccine hesitancy in some communities, which has also been observed in previous studies [38].

This assessment is subject to numerous limitations. First, the interview guides were developed in French. While some interviews with senior health workers were done in French, those with direct service providers at the community level were conducted mostly in local languages. It is possible different framing of questions in different local languages may have produced inconsistent interpretations and variations in meaning. The transcripts were not reviewed by the respondents for accuracy or to give opportunities to make clarifications. Moreover, audio recordings of interviews conducted in local languages may have been inconsistently translated during the transcription process. To mitigate these possible limitations regarding language, translation, and transcription, data collectors were trained on local translations of the interview guide. All data collectors were from Burkina Faso and fluent in the local languages of the regions where they were assigned. Using the same data collectors in translating and transcribing the audio recordings likely retained consistency in interpretations. Consistent with other qualitative approaches, these findings may not be generalizable to the wider population of health workers in Burkina Faso given the limited number of interviews conducted. However, multiple districts with varying vaccination coverage and health workers at multiple levels were purposively included to gain insights for recommendations to strengthen vaccination coverage via the EPI in Burkina Faso, especially as it pertains to MACV and MCV2. The number of interviewers mostly comprised of regional and district level managers compared to frontline workers. Nevertheless, we did not give greater weight to the number of responses obtained from each category of respondents; we instead focused our interpretation of themes based on variation in responses to show wide range of perspectives across multiple layers of the health system in Burkina Faso. Given the focus of the assessment, it is possible that interviewer bias may have resulted in a discourse that overly emphasized vaccination barriers. While interviewers’ familiarity with the local context was important to the success of the assessment, it is possible that certain assumptions were made based on tacit knowledge of the local setting. Finally, the results from the content analysis were never shared with respondents to get their feedback. However, the preliminary results were shared with other health officials from the Burkina Faso Ministry of Health as part of a dissemination workshop. Feedback received from the workshop informed our interpretation of the results and the discussion section. Finally, local knowledge of the issues may have biased our understanding and interpretation of the data obtained. However, local knowledge of complex issues may also be seen as a strength because we were able to carefully interpret nuanced issues that a ‘true outsider’ may not have fully grasped.

## Conclusions

Overall, health workers perceived that MACV was a source of motivation for caregivers to bring their children for the 15- to 18-month visit when MCV2 is also administered. However, service delivery barriers related to MACV and MCV2 co-administration need more attention. To identify opportunities to equitably increase coverage of MACV and MCV2 in Burkina Faso, perceptions of emerging community demand barriers reported by health workers in this assessment must be further examined. Alongside overall strengthening of service delivery systems, clear policy communication and implementation is needed to ensure that health workers do not refrain from opening multi-dose vials for small numbers of children; this could help improve missed opportunities for vaccination for both MCV2 and MACV. Health workers should benefit from an increased appreciation that vaccination in second year of life is an integral part of health services and build inter-personal communication skills to address socio-cultural beliefs and improve caregiver awareness of the need for timely and up-to-date vaccination. Other suggested system strengthening interventions include improving coordination across levels of the health system; strengthening defaulter tracking systems to reduce missed opportunities for vaccination after one year of age; assuring an adequate cold chain and supply of vaccines; and supporting transportation for outreach activities.

In general, effective administration of vaccines in children’s second year of life requires adequate operational and programmatic planning, training, communication, and monitoring.

## Supplementary information

**Additional file 1.** Supplemental Material: Health workers’ perceptions and challenges in implementing meningococcal serogroup A conjugate vaccine in the routine childhood immunization schedule in Burkina Faso.

## Data Availability

Data used for this study is available upon reasonable request and subject to a data use agreement. Data access requests should be sent to Dr. Heidi M. Soeters (hmsoeters@cdc.gov).

## Abbreviations

CHW: Community Health Worker
DPT: Diphtheria-Pertussis-Tetanus vaccine
EPI: Expanded Program of Immunization
IDI: In-depth interview
MACV: meningococcal serogroup A conjugate vaccine
MCV1: first dose of measles-containing vaccine
MCV2: second dose of measles-containing vaccine
MoH: Ministry of Health
NmA: *Neisseria meningitidis* serogroup A
UNICEF: United Nations Children’s Fund
WHO: World Health Organization
