# Supplementary material for: Health workers’ perceptions and challenges in implementing meningococcal serogroup a conjugate vaccine in the routine childhood immunization schedule in Burkina Faso
Source: BMC Public Health. 2020 Feb 19;20:254. doi: 10.1186/s12889-020-8347-z (PMC7031928; doi:10.1186/s12889-020-8347-z)
Supplement: Supplementary file 1 — Additional file 1. Supplemental Material: Health workers’ perceptions and challenges in implementing meningococcal serogroup A conjugate vaccine in the routine childhood immunization schedule in Burkina Faso. [file 12889_2020_8347_MOESM1_ESM.pdf]

Supplemental Material: Health workers' perceptions and challenges in implementing meningococcal serogroup A conjugate vaccine in the routine childhood immunization schedule in Burkina Faso

GUIDE: In-depth interviews with immunization staff

INTRO & OPENING STATEMENT

Thank you for taking the time to join us for this interview. I am ________, your interviewer, and this is ______ who will be taking notes as we talk and we will also be recording.

We hope for frank and open discussion where there are no right or wrong answers, everyone's perspective is valued. The discussion will take around 1-2 hours. Do you have any questions?

Perceptions of meningitis and measles

1. What are people's general understanding of meningitis and measles in this community? (*probe separately for each disease*)
    a. Probe: How much of a problem do people think it is?
    b. Probe: Who does it affect the most, and how severe is the disease?
    c. Probe: What do people usually do when it is suspected?
    d. Probe: What do people do to prevent it?

Information sources

2. How do caregivers learn about the 15-month visit for MenA and RR2 vaccination for children in this community?
    a. Probe: What are caregivers usually told; by whom and when?
    b. Probe: What are the promotion and engagement strategies around the introduction of MenA? How are these different or similar for what was done for RR2?
    c. Probe: Which social mobilization strategies do you find most effective for your community, and why?

Barriers

3. In general, what are the main reasons why some children do not receive all their vaccines at the scheduled time?
    a. Probe: Please tell us more about access issues
    b. Probe: Please tell us more about availability of vaccines or stock-outs
    c. Probe: Please tell us more about caregiver's confidence in vaccines
    d. Probe: Please tell us more about the community's awareness/interest in vaccines

4. What are the main reasons why some caregivers do not bring their children to the health facility for the 15-month visit for MenA and RR2 vaccination? How is this similar to or different from other routine vaccinations?
    a. Probe: Discuss distance to the health facility
    b. Probe: Discuss cost or payment
    c. Probe: Discuss trust in immunization staff
    d. Probe: Discuss religious and cultural factors

5. What are the top 3 challenges you face when with administering MenA vaccination?
    a. Probe: overall challenges with attendance at 15-month immunization visit?
    b. Probe: caregivers understanding of need for 15-month immunization?
    c. Challenges to providing MenA or RR2 vaccinations?

Motivations

6. Probe: How has the availability of MenA vaccination influenced caregivers' decision to bring their children for the 15-month visit?
    a. Probe: what can be done to encourage caregivers to bring their children for the 15-month vaccination visit where they also receive RR2?
    b. Probe: please give examples of novel experiences and/or successes in introducing and administering MenA at your health center

MenA and MCV2 Administration

7. How often do children receive MenA and RR2 at the same 15 month visit, and what are the reasons for not receiving both at the 15 month visit?
    a. Probe: Discuss caregiver refusal
    b. Probe: Discuss stock-out
    c. Probe: Discuss other relevant reasons

8. What do you think about the safety of co-administering MenA and RR2?
    a. Probe: from your point of view, are there concerns (rumors…) related to the coadministration of MenA and RR2? If so, what are they and how are they addressed?
    b. Probe: do caregivers have any concerns? If so, what are they and how are they addressed?

9. Are there any unwanted or unexpected results that you have observed regarding the introduction of MenA on RR2 services/uptake in your district? If so, please elaborate.

Recommendations

10. What strategies, if any, can the Ministry or this Health / Immunization Program employ to improve the proportion of children receiving all of their recommended vaccinations on time?
    a. What are your suggestions for helping children to catch up with their missed vaccinations, if needed?
    b. What other relevant recommendations do you have?

CLOSING STATEMENT

We have now reached the end of the interview. We would like to thank you for participating in today's interview. The information you shared is very important, and will help us better understand how to improve immunization services for children in your district.

Supplemental Material: Health workers' perceptions and challenges in implementing meningococcal serogroup A conjugate vaccine in the routine childhood immunization schedule in Burkina Faso

INFORMED CONSENT

Hello, my name is _____________________________, and I am joined today by colleague ____________________. We are here to talk to you about the health and wellbeing of children in this community. We would like to get your views and opinions on various issues that affect children in this community. The information you provide to you will help us improve the delivery of health services of children in this community.

Before we start the interview or discussion, I need to get your approval. Your participation is voluntary. The interview will be audio-recorded so that it can later be transcribed; and will be destroyed shortly after the transcription. Your name or other personal identifiable information will never be associated you're your responses in any published or other written reports. You're participating in the interview or discussion at your own free-will, and no one has coerced or forced you to do so. You do not have to respond to questions that you do not feel comfortable discussing. You can always exit the discussion should you decide to do so for any reasons.

If you have any questions or concerns regarding your participation in this interview you can freely contact:

Name ______________________________________
Roles ______________________________________
Tel: ______________________________________

CONSENT STATUS (verbal): _____Yes → *proceed with the interview or focus group discussion*

_____No → *END*

ANIMATEUR (must be completed for each interview or group discussion)

Full Name: (first, middle, last) ____

Signature ________________________ Date: _____________________

Supplemental Material: Health workers' perceptions and challenges in implementing meningococcal serogroup A conjugate vaccine in the routine childhood immunization schedule in Burkina Faso

Guide pour des entretiens approfondis avec le personnel de vaccination

INTRODUCTION & ALLOCUTION D'OUVERTURE

Merci d'avoir pris le temps de vous joindre à nous pour cette discussion de groupe. Je m'appelle ________, votre animateur, et voici ______ qui prendra des notes pendant que nous parlons ; et nous enregistrerons aussi cette discussion.

Nous espérons une discussion franche et ouverte où il n'y a pas de bonnes ou mauvaises réponses. On a besoin du point de vue de chacun. La discussion prendra environ 1-2 heures. Avez-vous des questions?

Perceptions de la méningite et de la rougeole

1. Quelle est la compréhension générale que les communautés ont de la méningite et de la rougeole dans votre aire de sante? (*sondez séparément pour chaque maladie*)
    a. Dans quelle mesure les communautés considèrent cette maladie comme étant un problème?
    b. D'après elles qui sont les plus affectés par la maladie? quelle est leur perception de la gravité de la maladie?
    c. Que font habituellement les communautés dans votre aire de sante si elles suspectent la maladie?
    d. Quelles sont les dispositions prises par la communauté pour prévenir la maladie?

Sources d'information

2. Comment les mères et les populations reçoivent l'information sur la visite de 15 mois pour la vaccination de MenA et RR2 pour les enfants de cette communauté?
    a. Que faites-vous de manière concrète pour tenir informé et susciter l'engagement des communautés à propos de la vaccination MenA? Avez-vous une stratégie différente ou similaire pour la RR2?
    b. Quelles stratégies de mobilisation sociale trouvez-vous le plus efficace pour votre communauté et pourquoi?

Barrières

3. En général, quelles sont les principales raisons pour lesquelles certains enfants ne reçoivent pas tous leurs vaccins au moment prévu?
    a. Parler nous davantage des problèmes d'accès (géographique, financière, culturelle…) au vaccin
    b. Quels sont les problèmes liés à la disponibilité des vaccins ou les ruptures de stock
    c. Quels sont les problèmes liés à la confiance qu'ont les communautés vis-à-vis des vaccins
    d. Quelles sont les difficultés liées à la sensibilisation et à l'engagement de la communauté pour les vaccins

4. Quelles sont les raisons principales pour lesquelles certaines personnes en charge n'amènent pas leurs enfants au centre de santé pour la visite de 15 mois pour la vaccination de MenA et RR2? En quoi est-ce que cette situation est similaire ou différente des autres vaccinations de routine?
    a. Question sonde: Parlons de l'accessibilité géographique Question sonde: Parlons du cout ou du paiement

   b. Question sonde: Parlons de la confiance envers le personnel de vaccination
   c. Question sonde: Parlons des facteurs religieux et culturels

5. Donnez-nous 3 principaux défis auxquels vous êtes confrontés lors de l'administration de la vaccination MenA?
   a. Les défis globaux liés à la participation à la visite de vaccination de 15 mois?
   b. Les mères ou tutrices comprennent-elles le besoin de la vaccination de 15 mois?
   c. Quels sont les défis relatifs à votre capacité d'offrir la vaccination MenA ou RR2?

Motivations

6. Comment la disponibilité de la vaccination MenA a-t-elle influencé la décision des personnes en charge d'amener leurs enfants pour la visite de 15 mois?
   a. Question sonde : Que peut-on faire pour encourager les mères et tutrices à amener leurs enfants à la visite de vaccination de 15 mois où ils reçoivent également la RR2?
   b. Question sonde : Quels ont été les expériences novatrices et ou succès dans le processus d'introduction et d'administration du MenA dans votre aire de santé?

L'administration de MenA et RR2

7. Est-ce qu'il est arrivé que des enfants n'aient pas pu recevoir à la fois la MenA et RR2 lors de la visite du 15ieme mois? quelles sont les principales raisons?
   a. Question sonde: Parlons du refus des mères et tutrices
   b. Question sonde: Parlons des ruptures de stock
   c. Question sonde: Parlons d'autres raisons pertinentes

8. Que pensez-vous de l'innocuité de la co-administration de MenA et RR2?
   a. Question sonde : De votre point de vue, est-ce qu'il y a eu des inquiétudes identifiées (rumeurs…) par rapport à la co-administration deux vaccins MenA et RR2 ? Si oui lesquelles, comment ont-elles été gérées?
   b. Question sonde: Les mères et tutrices ont-elles des préoccupations ? Si oui, quelles sont-elles et comment sont-elles traitées?

9. Y a-t-il des résultats indésirables ou inattendus (MAPI) que vous avez observés concernant l'introduction de MenA ou RR2 en termes de services / mise à jour dans votre district? Si oui, veuillez les décrire.

Recommandations

10. Quelles stratégies le ministère ou le programme de vaccination peut-il utiliser pour améliorer la couverture vaccinale selon le calendrier?
   a. Quelles sont vos suggestions pour aider les enfants à rattraper leur vaccination manquée, si nécessaire
   b. Quelles autres recommandations pertinentes avez-vous?

DISCOURS DE CLOTURE

Nous avons maintenant atteint la fin de notre discussion. Nous aimerions vous remercier d'avoir participé à la discussion d'aujourd'hui. Les informations que vous avez partagées sont très importantes et nous aideront à mieux comprendre comment améliorer les services de vaccination pour les enfants de votre district.

Supplemental Material: Health workers' perceptions and challenges in implementing meningococcal serogroup A conjugate vaccine in the routine childhood immunization schedule in Burkina Faso

Bonjour, je m'appelle _____________________________ et je suis rejoint aujourd'hui par un collègue ___________________. Nous sommes ici pour vous parler de la santé et du bien-être des enfants de cette communauté. Nous aimerions connaître votre point de vue et votre opinion sur diverses questions qui touchent les enfants de cette communauté. Les informations que vous nous fournissez nous aideront à améliorer la prestation des services de santé des enfants dans cette communauté.

Avant de commencer l'entretien ou la discussion, je dois obtenir votre approbation. Votre participation est volontaire. L'interview sera enregistrée en audio afin qu'elle puisse ensuite être transcrite; et sera détruit peu de temps après la transcription. Votre nom ou d'autres informations personnelles identifiables ne seront jamais associés à vos réponses dans les rapports publiés ou écrits. Vous participez à l'entretien ou à la discussion de votre plein gré, et personne ne vous a contraint ou forcé de le faire. Vous n'avez pas à répondre à des questions que vous ne vous sentez pas à l'aise de discuter. Vous pouvez toujours quitter la discussion si vous décidez de le faire pour quelque raison que ce soit

Nom ______________________________________
Désignation ______________________________________
Tel: ______________________________________

STATUT DE CONSENTEMENT (verbal):

_____Oui → procéder à l'entretien ou à la discussion de groupe
_____Non → FIN (merci pour leur temps)

Confirmation de l'animateur (doit être remplie pour chaque entrevue ou discussion de groupe)

"J'atteste avoir obtenu le consentement éclairé de l'interviewé ou des participants."

Nom: ____

Signature _______________________ Date: ____________________

Supplemental Material: Health workers' perceptions and challenges in implementing meningococcal serogroup A conjugate vaccine in the routine childhood immunization schedule in Burkina Faso

Debrief Notes

Interview Code (file name from audio recording):

**Date:**	**Start time:**	**End time:**
Name of facilitator/interviewer		
Name of note taker		
Region		
District		
Community/village		
Type of respondent		
Age of respondent		
Education of respondent		
Sex of respondent		
Years of experience in immunization		

Notes on the discussion

**Exact location of interview (example: in an office, house, under a tree..)**
**Mood of the discussion: Was the respondent talkative? Happy? Annoyed? Was there agreement/disagreement?**
**Personal reflections: what went well in the discussion? What could be improved?**

Supplemental Material: Health workers' perceptions and challenges in implementing meningococcal serogroup A conjugate vaccine in the routine childhood immunization schedule in Burkina Faso

**What was your Impression about the tool? And what do you think can be improved on the tool?**
**What were the main or most important themes and points that came out of the discussion?**

Supplemental Material: Health workers' perceptions and challenges in implementing meningococcal serogroup A conjugate vaccine in the routine childhood immunization schedule in Burkina Faso

Note de Synthèse

Code d'entrevue (nom du fichier de l'enregistrement audio):

**Date:**	**Heure de début:**	**Heure de fin:**
Nom de l'animateur		
Nom du preneur de notes		
Region		
District		
Communauté / village		
Type de répondant / groupe		

<u>Notes sur la discussion</u>

**Lieu exact de l'interview (exemple: dans un bureau, une maison, sous un arbre ..)**
**Humeur de la discussion: Le répondant était-il bavard? Content? Agacé? Y a-t-il eu accord / désaccord?**
**Réflexions personnelles: qu'est-ce qui s'est bien passé dans la discussion? Qu'est-ce qui pourrait être amélioré?**

Supplemental Material: Health workers' perceptions and challenges in implementing meningococcal serogroup A conjugate vaccine in the routine childhood immunization schedule in Burkina Faso

**Quelle a été votre impression sur l'outil? Et que pensez-vous peut être amélioré sur l'outil?**
**Quels ont été les thèmes ou les points principaux ou les plus importants qui sont ressortis de la discussion?**
